# Under-utilization of antenatal care services in Timor-Leste: results from Demographic and Health Survey 2009–2010

**DOI:** 10.1186/s12884-015-0646-5

**Published:** 2015-09-08

**Authors:** Vishnu Khanal, Jonia Lourenca Nunes Brites da Cruz, Shiva Raj Mishra, Rajendra Karkee, Andy H. Lee

**Affiliations:** Nepal Development Society, Bharatpur, Nepal; National Hospital Guido Valadares, Ministry of Health, Dili, Timor-Leste; School of Public Health and Community Medicine, BP Koirala Institute of Health Sciences, Dharan, Nepal; School of Public Health, Curtin University, Perth, Australia

## Abstract

**Background:**

Timor-Leste is a young country in the Asia-Pacific region with a high maternal mortality rate of 557 per 100,000 live births. As most maternal deaths can be prevented by providing quality antenatal care (ANC) and skilled assistance during childbirth, understanding the barriers to the utilization of ANC services can enhance program implementation. This study aimed to investigate the prevalence and factors associated with the under-utilization of ANC services in Timor-Leste.

**Methods:**

Timor-Leste Demographic and Health Survey (TDHS) 2009–2010 was a nationally representative multi-stage cross-sectional study involving 11,463 households and 9,828 childbirths. Information on last born child was recorded for 5,895 mother-child pairs. Factors influencing under-utilization of ANC were assessed using hierarchical logistic regression analysis.

**Results:**

Only 3311 (55.2, 95 % confidence interval (CI) 53.1 to 57.3 %) made the recommended four ANC visits, while 2584 (44.8; 95 % CI 42.7 to 46.9 %) of them reported attending three or less ANC services. Significant factors positively associated with the under-utilization of ANC were low wealth status (odds ratio (OR) 2.09; 95 % CI 1.68 to 2.60), no maternal education (OR 1.54; 95 % CI 1.30 to 1.82) or primary maternal education (OR 1.21; 95 % CI 1.04 to 1.41), no paternal education (OR 1.34; 95 % CI 1.13 to 1.60), and having a big problem in permission to visit health facility (OR 1.65; 95 % CI 1.39 to 1.96).

**Conclusions:**

Despite the apparently good progress made in re-establishing the healthcare infrastructure, 45 % of mothers remained in need of a focused intervention to increase their use of ANC services. Further prenatal care program should pay attention to women with low wealth status and those and their partners who are uneducated. Moreover, women should be encouraged to make decision on their own health.

## Background

Worldwide, almost 300,000 maternal deaths were reported in 2013 [1], with majority of these deaths occurred in developing countries. Maternal mortality ratio is 14 times higher in developing countries than in developed countries [2]. A large majority of these maternal deaths are preventable by providing quality antenatal care (ANC) and assisting child birth via skilled birth attendants. Maternal mortality has decreased globally by 1.3 % per year since 1990 [1], largely due to a better access to maternal health care. In addition to preventing maternal deaths, it is estimated that 12 % of neonatal deaths could be averted through ANC at 90 % coverage [3]. Attendance of ANC visits during pregnancy is associated with a higher use of institutional delivery service in India, Nepal and other developing countries [4–6]. It provides a platform for services whereby the health of woman is maintained during pregnancy, and improves pregnancy outcomes by identifying and managing pregnancy related complications [5]. Women receive information on birth spacing, fetal growth and development, as well as tetanus immunization at their ANC visits. Moreover, ANC contributes towards the prevention and treatment of malaria, management of anemia, and screening and treatment of sexually transmitted infections [7]. Therefore, the World Health Organization (WHO) has recommended at least four focused ANC visits during pregnancy [8].

Timor-Leste (East Timor) is a young country in the Asia-Pacific region which suffered a long conflict in the 1990s [9], leading to the destruction of most of its infrastructure, in addition to 200,000 deaths [10] and tens of thousands of its citizens being internally displaced [11]. The country declared its independence in 2002. Despite a shortage of trained health workforce in the post conflict era, the government has since re-established health services with the support from the United Nations, expatriate workers, and the Cuban training programs of medical professionals [12, 13]. Timor-Leste has a population growth rate of 2.4 % with 1.07 million inhabitants in 2010 [14]. Three quarters (73.5 %) of its population live in rural areas. The country has a high maternal mortality rate of 557 per 100,000 live births [15]. In addition to constructing new health facilities, the Timorese government has started a community based program to increase the utilization of ANC services. This program is known as “Servisu Integrado Sude Communita” (SISCa) [16] or ‘integrated health service program in the community’, which provides ANC services through outreach clinics, together with support from local community volunteers to encourage mothers to attend such services.

A large number of studies have investigated factors influencing the use of ANC services in developing countries. For instance, maternal education, parity, maternal autonomy (decision making on her own health), maternal occupation, partner’s education, distance to the health facility, and wealth status are known to be associated with the use of ANC [17–19]. Findings from the literature have also suggested that barriers to the utilization of ANC are contextual and may vary across cultures. Understanding such barriers is essential to increase the usage of ANC, in order to minimise any adverse pregnancy outcome. In the case of Timor-Leste, evidence is still lacking on the utilization of ANC services. Although there has been a significant increase in at least one ANC visit from 42.5 % in 2001 to 86 % in 2009 [15], information on attending the four recommended visits remains limited. Therefore, this study aimed to investigate the prevalence and factors associated with the under-utilization of ANC services in Timor-Leste. Results are potentially important for national managers to further monitor and improve the current SISCa maternal health program.

## Methods

This study used the dataset from the Timor-Leste Demographic and Health Survey (TDHS) 2009–2010, which was the second survey conducted since independence. The TDHS was a two stage cluster survey covering the entire nation of 1,163 enumeration areas. During the first stage, 455 enumeration areas were selected by probability sampling proportionate to size. A fixed number of 27 households were then randomly selected from each enumeration area [14]. The national survey has ethics approval from ICF Macro International and Ministry of Health, Timor-Leste [14]. Participants gave consent for themselves whereas mothers or care takers provided consent for minors including infants. The data collection process adhered to World Health Organization’s ethical and safety recommendations ([14], p. 227). The first author applied to the measureDHS and obtained permission to use the publicly available data for research and teaching learning purpose. Further details can be obtained from the website of measureDHS (https://dhsprogram.com/data/Using-DataSets-for-Analysis.cfm).

The TDHS 2009–2010 collected data from 11,463 households representative of the entire nation. The overall response rate was 93.5 %. In total, 13,137 women and 4076 men aged 15–49 years were interviewed. The survey used three sets of validated questionnaire (household, men, women) to collect information and the data were subsequently merged in separate datasets for further analyses. The *Childrenrecode* dataset [20] recorded all 9828 childbirths that occurred within the past five years (URL: http://dhsprogram.com/data/Dataset-Types.cfm). The present study included 5,895 mother-child pairs concerning the last born child only. Two missing and 38 ‘do not know’ cases were subsequently removed from the analyses.

**Fig. 1:**
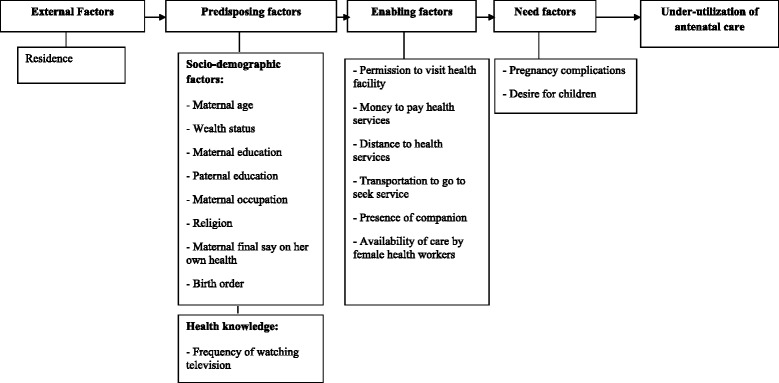
Conceptual framework of factors associated with under-utilization of antenatal care services in Timor-Leste. Adapted from Titaley et al. [21]

Figure 1 presents the underlying conceptual framework. The outcome variable was ‘under-utilization of ANC’, i.e. coded 1 if < 4 ANC visits and coded 0 if ≥ 4 ANC visits were made. Independent variables were selected according to the literature and their availability from the database [21], and were grouped into: external factors, pre-disposing factors (socio-demographic and health knowledge factors), enabling factors and need factors [6]. The sole external factor considered was residential location (rural versus urban). Socio-demographic factors included: maternal age (≤19 years; 20–34 years; and 35–49 years), wealth status (rich/upper 20 %; middle 40 %; and lower 40 %) [22]; maternal and paternal education (no education; primary school; secondary or higher education), maternal occupation (not working; agriculture; paid job: professional, clerical, business, service; manual job); religion (Roman Catholic; others: Muslim, Protestant, Hindu); maternal final say (decision) on her own health (herself; herself and others; others only); and birth order (rank) (1; 2 to 3; ≥ 4). The health knowledge factor was taken to be the frequency of watching television (not at all; less than once a week; at least once a week) following previous studies. Enabling factors were whether there was a ‘big problem’ (major problem) in (a) permission to visit health facility; (b) money to pay for health services; (c) distance to health services; (d) transportation to seek service; (e) presence of companion; and (f) availability of care by female health workers [21]. Need factors referred to pregnancy complications (severe abdominal pain, severe headache, convulsion, blurred vision and swelling of hands and feet, vaginal bleeding, and other problems that required immediate care) which were classified as either no complication or with complications. Desire for children was recoded into: wanted then; wanted later; and unwanted.

Statistical analyses were performed using the Statistical Package for Social Sciences (SPSS version 20). Data were first summarized by cross tabulation and chi-square tests. Number of ANC visits was presented as frequency distributions and corresponding 95 % confidence intervals (CI). Logistic regression analyses were then undertaken to ascertain the associations between under-utilization of ANC and the aforementioned factors, with adjusted odds ratios (OR) to assess the magnitude of such associations.

A hierarchical modelling strategy was adopted [21–23], with external factors initially entered into the logistic regression model as shown in Fig. 1. Socio-demographic factors were then entered into the model, followed by health knowledge factors, along with significant external and socio-demographic factors. In the final step, enabling factors were added to the model, as the need factors were deemed non-significant according to univariate chi-square tests. Complex sample analyses technique was adopted throughout to account for the study design and sample weight [24].

## Results

Table 1 presents the sample characteristics (*n* = 5895). The majority (75.4 %) of participants came from rural areas. Only a small proportion (2.9 %) of mothers aged ≤ 19 years. One in five mothers (20.8 %) had higher socioeconomic status, while about one third (32.8 %) received no education. Less than a quarter of mothers could decide herself with regard to seeking health care (23 %) or watched television daily (24 %). A vast majority (94.3 %) of them experienced at least one pregnancy complication: severe abdominal pain (37.0 %), severe headache (42.4 %), convulsion (47.1 %), blurred vision and swelling of hands and feet (11.7 %), vaginal bleeding (5.7 %), and other problems that required immediate care (26.3 %).

**Table 1:** Characteristics of participants and association with under-utilization of antenatal care, Timor-Leste

Factors	*n* (%)	ANC ≥ 4	ANC <4^a^	P*
External Environment				
Residence				<0.001
Urban	1351 (24.6)	821 (28.2)	530 (20.2)	
Rural	4544 (75.4)	2490 (71.8)	2054 (79.8)	
Predisposing Factors				
Socio-demographic				
Maternal age (years)				<0.001
15–19	185 (2.9)	103 (2.8)	82 (3.0)	
20–34	3518 (60.5)	2066 (63.4)	1452 (56.9)	
35–49	2192 (36.6)	1142 (33.8)	1050 (40.1)	
Wealth status				<0.001
Low (lower 40 %)	2512 (39.9)	1141 (31.2)	1371 (50.7)	
Middle (middle 40 %)	2407 (39.3)	1499 (43.0)	908 (34.7)	
High (upper 20 %)	976 (20.8)	671 (25.8)	305 (14.6)	
Maternal education				<0.001
No education	1980 (32.8)	904 (26.6)	1076 (40.5)	
Primary	189 (27.5)	942 (26.9)	747 (28.3)	
Secondary or higher	2226 (39.6)	1465 (46.6)	761 (31.1)	
Paternal education				<0.001
No education	1658 (27.9)	748 (22.2)	910 (35.1)	
Primary	1668 (27.4)	945 (27.5)	723 (27.3)	
Secondary or higher	2558 (44.6)	1613 (50.3)	945 (37.6)	
Maternal occupation				<0.001
No paid work	3536 (61.0)	1965 (59.7)	1571 (62.7)	
Agriculture	1551 (24.5)	813 (23.1)	738 (26.2)	
Paid work (professional, clerical, sales, service, business)	675 (11.9)	445 (14.1)	230 (9.2)	
Paid work (manual)	125 (2.6)	85 (3.1)	40 (1.9)	
Religion				0.597
Roman Catholic	5770 (98.0)	3247 (98.1)	2523 (97.8)	
Others	125 (2.0)	64 (1.9)	61 (2.2)	
Maternal final say on her own health care				0.388
Herself	1391 (23.0)	770 (22.9)	621 (23.2)	
Herself and others	3610 (64.2)	2076 (65.0)	1534 (63.2)	
Others	683 (12.8)	372 (12.1)	311 (13.6)	
Birth order				<0.001
1	853 (14.3)	517 (15.5)	336 (12.9)	
2–3	1655 (29.1)	969 (30.6)	686 (27.2)	
≥4	3387 (56.6)	1825 (53.9)	1562 (60.0)	
Health knowledge				
Frequency of watching television				<0.001
Not at all	3743 (59.9)	1913 (53.0)	1830 (68.4)	
Less than once a week	516 (8.4)	323 (9.2)	193 (7.5)	
At least once a week	1636 (31.7)	1075 (37.9)	561 (24.1)	
Enabling Factors				
Permission to visit health facility				<0.001
Big problem	1379 (22.3)	646 (18.2)	733 (27.4)	
Not a big problem	4515 (77.7)	2665 (81.8)	1850 (72.6)	
Money to pay for health services				<0.001
Big problem	2266 (35.1)	1108 (30.1)	1158 (41.2)	
Not a big problem	3629 (64.9)	2203 (69.9)	1426 (58.8)	
Distance to health facility				<0.001
Big problem	3493 (56.0)	1811 (51.1)	1682 (62.1)	
Not a big problem	2400 (44.0)	1498 (48.9)	902 (37.9)	
Transportation to go to seek service				<0.001
Big problem	3625 (61.6)	1892 (57.2)	1734 (67.1)	
Not a big problem	2269 (38.4)	1419 (42.8)	850 (32.9)	
Presence of companion				0.002
Big problem	2594 (42.5)	1361 (40.3)	1233 (45.3)	
Not a big problem	3301 (57.5)	1950 (59.7)	1351 (54.7)	
Availability of care by female health workers				0.004
Big problem	3844 (64.1)	2101 (61.9)	1743 (66.7)	
Not a big problem	2051 (35.9)	1210 (38.1)	841 (33.3)	
Need Factors				
Pregnancy complications				0.451
No complication	332 (5.7)	181 (6.0)	151 (5.4)	
With complications	5563 (94.3)	3130 (94.0)	2433 (94.6)	
Desire for children				0.993
Wanted then	5000 (84.4)	2816 (84.4)	2184 (84.3)	
Wanted later	676 (12.3)	376 (12.3)	300 (12.3)	
Unwanted	219 (3.3)	119 (3.3)	100 (3.4)	

*Chi-square test of association

^a^Under-utilization of ANC

Table 2 gives the frequency distribution of number of ANC visits. Among the 5895 mothers, 2584 were attending three or less ANC services, giving a prevalence of 44.8 % (95 % CI 42.7 to 46.9 %) for under-utilization. Table 1 further shows the univariate associations between the outcome variable and each independent variable, whereas Table 3 presents the set of significant influencing factors in the final logistic regression model. Significant risk factors for under-utilization of ANC were low wealth status (OR 2.09; 95 % CI 1.68 to 2.60), no maternal education (OR 1.54; 95 % CI 1.30 to 1.82) or primary maternal education (OR 1.21; 95 % CI 1.04 to 1.41), no paternal education (OR 1.34; 95 % CI 1.13 to 1.60), and having a big problem in permission to visit health facility (OR 1.65; 95 % CI 1.39 to 1.96).

**Table 2:** Frequency distribution of antenatal care visits, Timor-Leste (*n* = 5,895)

ANC visits	Number	Percent (95 % CI)
0	738	12.6 (11.3, 14.0)
1	200	3.2 (2.7, 3.8)
2	680	11.8 (10.7, 13.0)
3	966	17.2 (15.9, 18.6)
≥4	3311	55.2 (53.1, 57.3)

**Table 3:** Factors associated with under-utilization of antenatal care in Timor-Leste

Factors	Crude odds ratio (95 % CI)	Adjusted odds ratio (95 % CI)	*P*
Wealth status			<0.001
High (upper 20 %)	1.00	1.00	
Low (lower 40 %)	2.89 (2.36, 3.52)	2.09 (1.68, 2.60)	
Middle (middle 40 %)	1.43 (1.19, 1.73)	1.18 (0.96, 1.44)	
Maternal education			<0.001
Secondary or higher	1.00	1.00	
No education	2.28 (1.96, 2.66)	1.54 (1.30, 1.82)	
Primary	1.58 (1.36, 1.83)	1.2 (1.04, 1.41)	
Paternal education			0.001
Secondary or higher	1.00	1.00	
No education	2.11 (1.80, 2.48)	1.34 (1.13, 1.60)	
Primary	1.32 (1.14, 1.54)	1.00 (0.86, 1.17)	
Permission to visit health facility			<0.001
Not a big problem	1.00	1.00	
Big problem	1.70 (1.42, 2.03)	1.65 (1.39, 1.96)	

## Discussion

This study found that 45 % of the mothers did not attend the recommended four ANC services; among them, 12.6 % reported that they had made no ANC visits. Being a newly independent country, Timor-Leste has an inadequate infrastructure and limited human resources which may partly attribute to the apparently low utilization of ANC services [25]. The country has been implementing its community-based programs such as integrated community health service (SISCa) and family health promoters (‘Promotor Saude Familia’) [26]. The promotion of usage of maternal health services remains a high priority for the Timor-Leste government and future TDHS may be able to evaluate the effect of such efforts. Decision making power of mothers is found to be a significant factor influencing health service utilization. Not having such power and autonomy of decision has been reported as a major barrier in the utilization of maternity services in Indonesia [21] and Ethiopia [27]. The overall patriarchal society in Timor-Leste and low level of maternal education might have contributed to their lack of household decision making, resulting in a high rate of non- utilization of ANC services. Several maternal health promotion programs in developing countries have focused on motivating mothers to attend maternal health services [16, 19]. However, mothers generally do not have the power to make decision. The current finding therefore indicates the need to educate family members to create an appropriate environment so that mothers can make decision on service utilization by themselves [19]. The Timorese Ministry of Health has commenced the “Promotion of Equality Strategy” [15] to support gender sensitive policies. Our finding also provides evidence to support such an initiative.

We found that mothers from households with lower wealth status being at a higher risk of not making the recommended four ANC visits, consistent with national surveys conducted in Indonesia where mothers from poor households tended to be less likely to attend ANC services [21]. Economic hardship may restrict mothers to travel, access and pay for the expenses associated with the use of such services [19]. In addition, they will lose their working hours to earn income which might have a large impact on their livelihood. Recently, financial hardship has been identified as the main reason behind non-compliance to attend ANC [26]. Provision of transportation cost, similar to those implemented in the ‘safer mother programme’ of Nepal [28], may help to overcome the problem in Timor-Leste.

Influence of parental education on ANC service utilization is well documented [19]. This study similarly found that those mothers with little education and whose partners had no education were less likely to attend the recommended four ANC visits. In general, educated parents have better knowledge on the importance of ANC and ability to decide when and where to seek health care, together with financial resources [29].

Findings of this study are useful for setting a baseline for SISCa [16] and to identify those women who under-utilized the ANC services, so that family health promoters can be mobilized [26] at the national level and the next THDS would provide an overall progress in antenatal service. The results suggest targeting those who are poor, less educated, and who are not able to make self-decision for seeking services. If mothers cannot visit the service centres, they should be made aware that current SISCa [16] can bring services to their community. Moreover, health education including knowledge of danger signs of pregnancy and delivery should be advocated, as birth preparedness programs have been found effective in promoting the use of ANC services and subsequent institutional delivery [28].

The present study provides the first report using nationally representative data. The findings have important implications for planning of future studies in maternal health. A major limitation concerns the cross-sectional study designs which was based on recall and self-report. Nonetheless, such methods have been shown to be successful and effective for developing countries whose national source of data is derived from surveys of this nature and magnitude.

## Conclusion

About 45 % of Timorese mothers did not make the recommended four ANC visits. Women with low wealth status, as well as those and their partners who are uneducated, should be targeted to increase their participation in ANC promotion programs. Moreover, women should be encouraged to make decision on their own health via existing community based programs such as SISCa and family health promoters. In the long run, empowering the overall status of women through education would increase their utilization of ANC services.
